# Effect of Temperature and Maternal Age on Recombination Rate in Cattle

**DOI:** 10.3389/fgene.2021.682718

**Published:** 2021-07-20

**Authors:** Botong Shen, Ellen Freebern, Jicai Jiang, Christian Maltecca, John B. Cole, George E. Liu, Li Ma

**Affiliations:** ^1^Department of Animal and Avian Sciences, University of Maryland, College Park, College Park, MD, United States; ^2^Department of Animal Science, North Carolina State University, Raleigh, NC, United States; ^3^Animal Genomics and Improvement Laboratory, BARC, USDA-ARS, Beltsville, MD, United States

**Keywords:** recombination, maternal age, temperature, cattle, genetics

## Abstract

Meiotic recombination is a fundamental biological process that facilitates meiotic division and promotes genetic diversity. Recombination is phenotypically plastic and affected by both intrinsic and extrinsic factors. The effect of maternal age on recombination rates has been characterized in a wide range of species, but the effect’s direction remains inconclusive. Additionally, the characterization of temperature effects on recombination has been limited to model organisms. Here we seek to comprehensively determine the impact of genetic and environmental factors on recombination rate in dairy cattle. Using a large cattle pedigree, we identified maternal recombination events within 305,545 three-generation families. By comparing recombination rate between parents of different ages, we found a quadratic trend between maternal age and recombination rate in cattle. In contrast to either an increasing or decreasing trend in humans, cattle recombination rate decreased with maternal age until 65 months and then increased afterward. Combining recombination data with temperature information from public databases, we found a positive correlation between environmental temperature during fetal development of offspring and recombination rate in female parents. Finally, we fitted a full recombination rate model on all related factors, including genetics, maternal age, and environmental temperatures. Based on the final model, we confirmed the effect of maternal age and environmental temperature during fetal development of offspring on recombination rate with an estimated heritability of 10% (*SE* = 0.03) in cattle. Collectively, we characterized the maternal age and temperature effects on recombination rate and suggested the adaptation of meiotic recombination to environmental stimuli in cattle. Our results provided first-hand information regarding the plastic nature of meiotic recombination in a mammalian species.

## Introduction

Meiotic recombination is an essential process that occurs in all sexually reproducing organisms. This process facilitates the pairing and alignment of homologous chromosomes during prophase, which leads to the formation of crossovers. These crossover events transfer genetic information between the maternal and paternal homologs, and in doing so, ensures that each offspring will receive a unique combination of parental genomes. The extent and pattern of genetic reshuffling has important implications for evolution and population genetics, as well as breeding. However, errors in meiotic recombination can lead to aneuploidy, chromosomal abnormalities, and other deleterious outcomes (Hassold and Hunt, 2001; Lipkin et al., 2002). Thus, meiotic recombination must be well-regulated by cellular processes to prevent disturbances in the recombination pathway. It has been found that various factors influence meiotic recombination patterns in human and animal genomes. For instance, genome-wide association studies (GWAS) have identified genes and genetic variants associated with recombination features in humans (Kong et al., 2008; Chowdhury et al., 2009), mice (Baudat et al., 2010), cattle (Sandor et al., 2012; Ma et al., 2015; Shen et al., 2018), and sheep (Johnston et al., 2016). Some of the genes from those studies, including *RNF212*, *CPLX1*, and *PRDM9*, have been reported to be associated with individual-level recombination rates across multiple mammalian species.

A variety of intrinsic and external factors affect recombination rates across individuals and populations. These factors can be derived from environmental conditions, such as temperature, or physiological and stressful conditions, such as starvation. The resulting changes in recombination rates pose benefits or consequences to the health and evolution of a species. Many studies have suggested that maternal age’s intrinsic factor has a significant effect on recombination rate, but the direction of the effect is still debatable (Polani and Jagiello, 1976; Hussin et al., 2011; Martin et al., 2015). A recent multicohort analysis in humans reported a small but significant positive effect of maternal age on recombination rate, which contradicted previous studies in other human population (Martin et al., 2015). In mice and hamsters, a negative effect of maternal age was observed (Polani and Jagiello, 1976). However, no effect of maternal age was found for recombination rate in wild sheep (Johnston et al., 2016), but an increase was reported in swine (Lozada-Soto et al., 2021). Also, extensive studies of the maternal age effect have been conducted in *Drosophila*, worms, plants, and yeast, but no consistent conclusion have been reached (Hunter et al., 2016; Modliszewski and Copenhaver, 2017). As for the paternal side, many studies reported no effect of paternal age on meiotic recombination (Griffin et al., 1995; Hussin et al., 2011). Although the biological reason remains unclear for the effect of maternal age on recombination, there are some proposed explanations for both directions of the effect. The positive effect can be explained by a selection hypothesis: the factors related to aneuploidies increase with maternal age, so eggs with more crossovers are more likely to overcome these and give a live birth in older mothers (Kong et al., 2004). The negative effect can be explained by another hypothesis that specific meiotic configurations are less likely to be properly processed with increasing maternal age, so recombination rate decreases with maternal age (Hassold et al., 1995).

Extrinsic factors, such as temperature and nutrient conditions, have also been found to influence meiotic recombination rates. In *Drosophila*, environmental stressors such as exposure to Ethylenediaminetetraacetic acid (EDTA) or nutritional deficiency were observed to increase the recombination rate (Levine, 1955). Similarly, in the budding yeast, *Saccharomyces cerevisiae*, a lack of nutritional resources resulted in an increased recombination rate (Abdullah and Borts, 2001). However, the effect of temperature on meiotic recombination rates is more complicated. Some studies reported a positive correlation in *Arabidopsis thaliana*, *Caenorhabditis elegans*, and *Melanoplus femurrubrum* (Church and Wimber, 1969; Rose and Baillie, 1979; Francis et al., 2007), while other studies found a negative correlation in *Allium ursinum* (Loidl, 1989).

Additionally, both positive and negative correlations were detected in *Drosophila* (Stern, 1926). For instance, a recent study in *Drosophila melanogaster* reported a non-linear increase in meiotic recombination frequency in response to increased exposure to heat shock conditions (Jackson et al., 2015). This finding suggests that *Drosophila* can plastically modulate their recombination rate in response to environmental conditions, thus conferring greater adaptive potential to their offspring. In cattle, decreases in fertility rate have occurred due to the major factor of heat stress. In fact, Holstein cattle’s conception rate in the summer season is 20–30% less than in the winter season (Cavestany et al., 1985).

The formation of the Animal Genomics and Improvement Laboratory (AGIL) has facilitated the development of genetic evaluations for economically important traits in United States dairy cattle. Such studies can enhance research to improve the health and efficiency of cattle, including the study of recombination features across multiple cattle breeds with high statistical power. Using the large cattle genomic database maintained by AGIL and the Council on Dairy Cattle Breeding (CDCB), we have previously characterized the recombination features and their genetic control in dairy cattle. As mounting evidence has shown, meiotic recombination rates respond to both intrinsic and extrinsic conditions. Therefore, this study aims to determine how recombination rates vary in relation to advancing maternal age and common environment factors, such as temperature.

## Results and Discussion

### Identification of Recombination Events in Genotyped Cattle Pedigree

Using a method developed in our previous studies (Ma et al., 2015; Wang et al., 2016), we identified recombination events by constructing three-generation families from a large cattle pedigree that included an offspring, parents, and grandparents. We phased the SNP genotypes of the focal offspring and its parents within each three-generation family. We then inferred maternal crossover events by comparing phased genotypes between dam-offspring pairs. To ensure optimal data quality, we excluded the X chromosome and used the SNP coordinates that have been corrected for potential mapping errors (Null et al., 2019; Rosen et al., 2020). In total, we extracted 305,545 three-generation families and identified 6,677,618 maternal crossover events. All the animals have birth dates available, so we used the age of parent at birth of the focal offspring to study the effect of maternal age. Farm location and temperature information were available for 36,999 parents, which were included in the temperature effect analysis.

### Effects of Maternal Age on Recombination Rate in Cattle

**FIGURE 1:**
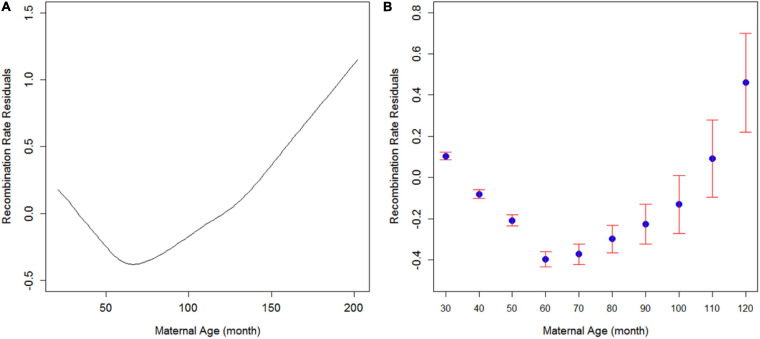
Trend of recombination rate residuals against maternal age in cattle. **(A)** Fitted smooth spline of recombination rate residuals along with maternal age. The smooth spline was fitted in R using the smooth spline function between recombination rate residuals and maternal age. **(B)** Recombination rate residuals in different maternal age groups. Blue dots are means, and bars are standard errors.

Previous studies have suggested a relationship between maternal age and the number of recombination events in plants, mice, and humans. However, even within the same species, the direction of this correlation remains inconsistent. To investigate how recombination rates are related to maternal age in cattle, we first modeled this relationship with a continuous variable of maternal age and the recombination rate residuals in cattle (Figure 1A). The recombination rate residuals were obtained by adjusting recombination rates with SNP chip density and the number of informative SNP markers within each of the 305,545 three-generation families. As a result, we observed a quadratic trend where recombination rate initially decreased from 20 to 65 months old parents and then increased as the cow grew older than 65 months. Note that we have more statistical support for the decreasing trend of recombination rate from 20 to 65 months since it consists of 91.8% of our records with much smaller standard errors. Parents that gave birth between 65 and 100 months old consist of 21,798 (7.1%) cases of our data, and we only have 3,321 (1.1%) cows giving birth above 100 months age. Still, the increasing trend after 65 months of maternal age is supported with a reasonable sample size (>25,000). This increasing trend of recombination rate in older parents is consistent with maternal age’s positive effect on recombination rate in the latest multicohort human study (Martin et al., 2015).

Since 65 months is the age that separated two groups of cows by the direction of maternal age effect, we divided the cows into ten age groups starting from 20 months old with an increment of 10 months and plotted the relationship (Figure 1B). Consistently, the same quadratic trend was observed when using maternal age as groups. Note that the last age group consists of all the records of parents giving birth over 120 months of age. To the best of our knowledge, this is the first such study in a mammalian species that reported a U-shaped relationship between maternal age and recombination rate. However, a quadratic relationship was also identified for the effect of temperature on the recombination rates in plants, fruit flies, and grasshoppers (Church and Wimber, 1969; Phillips et al., 2015), although the underlying mechanisms of meiotic recombination are completely different between cattle and these species. And this increasing of recombination after 65 months of age can also be due to culling and data representation issues because only the most fertile cows can survive on a farm for more than 65 months.

### Effects of Temperature on Maternal Recombination Rates in Cattle

Previous studies have shown that temperature affects meiotic recombination rates in many poikilothermic organisms, including yeast, plants, worms, grasshoppers, and large reptiles such as crocodiles (Church and Wimber, 1969; Isberg et al., 2006; Phillips et al., 2015). However, the direction of the effect remains inconclusive. Utilizing the extensive cattle pedigree data from the United States National Cooperators Database, we characterized meiotic recombination features in dairy cows and integrated them with the environmental temperature information. Using the NOAA National Weather Database, we obtained temperature data for the months when the calves were conceived by the parents of interest. In total, we have 36,999 records with both the environmental temperature and recombination rate data. We fitted a model to explore the temperature effect on maternal recombination rates in cattle. THI (temperature humidity index) has been widely used to indicate heat or cold stress in cattle (Gaughan et al., 2008). An environment with THI exceeding 78 can be considered as a heat stress condition for cattle because both the productive and reproductive performance of cows would be seriously affected (Bohmanova et al., 2007). There is no predetermined THI index for cold stress conditions as cattle’s wellness in a cold environment depends on several factors such as management practices and their hair coats. Based on the THI index in cattle, we chose two temperatures as the threshold of hot and cold conditions. Temperatures above 26.67°C were considered as hot conditions and temperatures below 4.44°C as cold conditions. In this study, we tested the effect of temperature rather than THI on recombination rate as most of previous studies reported the effect of temperature rather than THI (Church and Wimber, 1969; Loidl, 1989; Phillips et al., 2015). Still, it is interesting to investigate whether THI may have a larger impact than temperature only on recombination in future studies.

**FIGURE 2:**
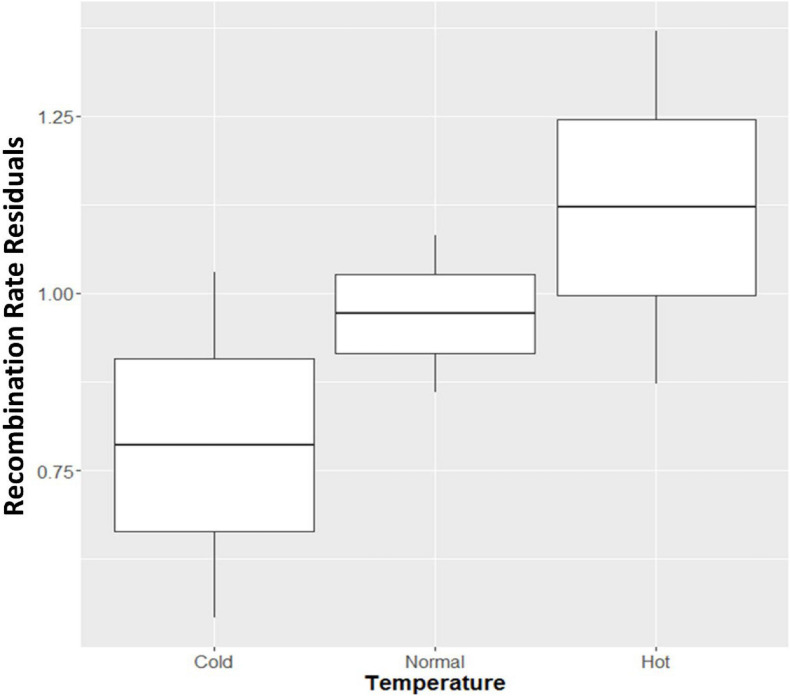
Boxplot of recombination rate residuals in three temperature conditions during the fetal development of offspring. **Cold:** temperatures below 4.44°C; **Normal:** temperatures between 4.44 and 26.67°C; **Hot:** temperatures above 26.67°C.

The cows were divided into three groups based on the temperature condition during the fetal development of the offspring. Over 6 K cows were conceived under hot conditions, over 25 K cows were conceived in a mild temperature environment, and 6 K cows were conceived under cold environment. To characterize the effect of this temperature, we reported box plots of the recombination rate residuals against those three conditions (Figure 2). We observed that cows under hot temperatures during pregnancy showed an elevation of recombination rate while cows under cold conditions showed decreased recombination rate. An increase of recombination events under hot environment is consistent with many previous studies across several species, which found an increase in recombination frequency under heat stress conditions (Lim et al., 2008; Jackson et al., 2015; Modliszewski and Copenhaver, 2017; Arrieta et al., 2021). However, the temperature effect identified here was for the fetal development stage of the offspring, instead of the female parent. Note that it is the fetal development stage of the female parent that is crucial for meiotic recombination in mammals. Therefore, the temperature effect reported here could possibly be due to an indirect effect on the fitness of the offspring with more or less crossovers, rather than on the meiosis process itself. Finally, the reported temperature effect can be explained by the ‘production line’ hypothesis for female recombination (Henderson and Edwards, 1968; Kong et al., 2004).

### Full Model Analysis of Recombination With Genetics, Maternal Age, and Temperature

To fully understand the effect of genetic and non-genetic factors on recombination, we fitted a full model on recombination rate with all relevant factors available in our data. We modeled the genetic or animal effect as a random effect and temperature condition as a fixed effect with three levels. We included the temperatures during two important developmental stages of a female meiosis, one during the fetal development for the offspring (first generation in a three-generation family) and the other during the fetal development of the parent (second generation). Finally, we also included maternal age, the parent’s birth year, and the quadratic term of these factors in the model (Table 1). Based on the genetic effect, we estimated the recombination rate’s heritability to be 10% (*SE* = 0.03), consistent with other studies in livestock animals (Sandor et al., 2012; Johnston et al., 2016; Zhang et al., 2020). Our results from this full model showed that hot temperature during the fetal development of the offspring would increase recombination rate (*P* = 0.027), while cold conditions were associated with decreased recombination rate (*P* = 0.019). However, the temperature (hot and cold conditions) during the fetal development of parents were not significantly associated with recombination rate (*P* = 0.271 and *P* = 0.097, respectively), although that is when female meiosis arrests. We also found that maternal age has a negative effect on recombination rates (*P* = 4.83 × 10^–12^) with a significant quadratic term (*P* = 5.68 × 10^–4^), confirming the U-shaped relationship between maternal age and recombination rate. We also noticed that a parent’s birth year would positively influence recombination rates (*P* = 1.51 × 10^–31^) with a significant quadratic term (*P* = 1.01 × 10^–40^), indicating either an increasing trend of recombination rate in the dairy cattle population or some inherent confounding in the data.

**TABLE 1:** Results of the mixed model analyses of eight factors related to the recombination rates in cattle.

**Factor**	**Beta**	***P*-value**
Cold temperature during fetal development of offspring	−0.194	0.019
Hot temperature during fetal development of offspring	0.167	0.027
Cold temperature during fetal development of parent	0.139	0.097
Hot temperature during fetal development of parent	0.093	0.271
Maternal Age	−0.082	4.83 × 10^–12^
Maternal Age^2^	4.69 × 10^–4^	5.68 × 10^–4^
Parent Birth Year	5.02 × 10^–3^	1.51 × 10^–31^
Parent Birth Year^2^	−4.41 × 10^–7^	1.01 × 10^–40^

### Potential Application of Recombination to Animal Breeding

Theoretically, recombination should be beneficial for the long-term efficiency of selection through increasing genetic variation (Otto and Barton, 2001). However, in a short-term period, recombination may also break the combination of beneficial alleles in the haplotypes that were selected for breeding. Recent simulation studies have shown that the effect of modifying recombination rate on the improvement of traditional genomic selection is small (Battagin et al., 2016). Still, our recent work on gamete variance provided another way of using recombination on short term selection, especially for bull sires and bull dams (Santos et al., 2019). The quadratic effect of maternal age on recombination rate suggests that young bull dams with higher recombination rate will have larger gametic variance and a better chance of producing eggs with extreme genetic merit. Finally, recombination rate does not need to be included as an independent trait in selective breeding because it has no direct economic values. But it will be under indirect, positive selection when breeding program is effective and proper selection indices used because of the long-term benefit of recombination in promoting genetic and gametic variations.

## Conclusion

It has been shown that recombination rate can fluctuate in response to environmental changes. In this study, we used large pedigree data of dairy cattle to test the association between recombination rate and genetic and non-genetic factors, including maternal age and temperature. We discovered a non-linear association between maternal age and recombination rate in cattle, which has not been described before. Additionally, we found elevated recombination rates with increasing environmental temperature during conception. Taken together, our study provides clear evidence of an association between meiotic recombination with the non-genetic factors of maternal age and temperature. These results reveal useful insights into both the intrinsic and extrinsic effects on meiotic recombination.

## Materials and Methods

### Estimation of Recombination Rate in Cattle Pedigree

We used an approach similar to the one that was developed in previous studies (Ma et al., 2015; Shen et al., 2018). First, we identified recombination/crossover events in genotyped cattle pedigree data from the national dairy genomic database hosted at the Council on Dairy Cattle Breeding (CDCB). Based on the millions of animals with genotype and pedigree data available, we extracted 305,545 three-generation families where an offspring (first generation), at least one parent (second generation), and at least one grandparent (third generation) were genotyped. We then phased the two haplotypes of an animal (first and second generations) based on the parental genotypes. We identified crossover events by comparing haplotypes in the first and second generations. Recombination events were assigned to an interval flanked by two informative SNPs (phased heterozygote SNPs in the second generation), and we estimated recombination rate between consecutive SNPs by the average crossover numbers per meiosis. We only used three-generation families that were genotyped by at least 50 K SNP chips. We used the ARS-UCD 1.2 genome assembly (Rosen et al., 2020) with updated SNP coordinates^1^ and removed the loci from problematic regions identified in previous studies (Null et al., 2019). We only used autosome data due to the quality issues with the sex chromosomes. We also removed animals with more than 45 genome-wide recombination events based on the distribution of recombination across all animals, which is close to a normal distribution with mean 23.2 and variance 98.3.

### Temperature Data From the NOAA Database

The National Oceanic and Atmospheric Administration (NOAA) is an American scientific agency that focuses on the conditions of the oceans and atmosphere. It’s also the largest database that contains the weather records of most United States cities since 1970s. By accessing the NOAA database using the R package “rnoaa” (Edmund et al., 2014), we extracted the weather conditions during two critical periods of a cow’s development that may affect the female meiotic recombination process (Table 1). The first temperature was the average temperature in the month prior to the birthdate of the offspring that measures the fetal development environment of the offspring (the first generation in a three-generation family). The second temperature was the average temperature during the month prior to the birthdate of the parent that measures the environment during the fetal development of the parent (the second generation). We then combined the temperature data with our recombination records for our mixed model analysis. By considering both the range of temperature and data availability, we divided the original temperature data into three levels: temperatures above 26.67°C are considered to be “hot,” temperatures below 4.44°C are considered as “cold,” and temperatures in between are “normal.”

### A Full Model Analysis of Genetics, Maternal Age, and Temperature

From each of the 36,009 three-generation families, we estimated the total number of crossover events per meiosis of the female parent (second generation). We then adjusted the number of crossover events by SNP density and the number of informative markers (phased heterozygote SNPs) of each animal. We first checked the maternal age effect using a smooth spline and boxplot. The smooth spline was fitted in R using the smooth spline function between recombination rate residuals and maternal age. Using the recombination rate residuals as phenotypes, we also fitted a linear mixed model to test for the effect of all available factors on recombination rate using the MMAP software (O’Connell, 2013). The model equation was fitted as following,

$$\text{Y}=\alpha +{\text{T}}_{1}+{\text{T}}_{2}+\text{A}+{\text{A}}^{2}+\text{B}+{\text{B}}^{2}+\text{g}+\varepsilon$$

where **Y** refers to the recombination rate residuals of individuals, **T_1_** and **T_2_** are the fixed effects for low and high temperatures, **A** represents a fixed effect of maternal age, **A^2^** represents the squared effect of maternal age, **B** and **B^2^** represents the fixed effect of parent’ birth year and its square, and **g** is a random effect for the genetic or animal effect on recombination rate with **g** ∼*N*(0, σ^2^**G**) and **G** being a genomic relationship matrix of the individuals calculated using the approach developed by VanRaden (2008). Both the temperatures during the fetal development of the parents and offspring were tested in this model. Statistical differences were declared as significant at *P* < 0.05.

## Data Availability Statement

The original contributions presented in the study are included in the article. Further inquiries can be directed to the corresponding author.

## Ethics Statement

Ethical review and approval was not required for the animal study because no live animals are used.

## Author Contributions

LM and CM conceived the study. BS, JJ, and EF analyzed and interpreted the data. EF, BS, and LM wrote the manuscript. GL and JC contributed the tools and materials. All authors read and approved the final manuscript.

## Conflict of Interest

The authors declare that the research was conducted in the absence of any commercial or financial relationships that could be construed as a potential conflict of interest.

## Footnotes

1 https://www.animalgenome.org/repository/cattle/UMC_bovine_coordinates/

## Abbreviations

SNP: single nucleotide polymorphism
Chr: chromosome
NOAA: National Oceanic and Atmospheric Administration
THI: Temperature humidity index.
